# Good and Bad Sides of Self-Compassion: A Face Validity Check of the Self-Compassion Scale and an Investigation of its Relations to Coping and Emotional Symptoms in Non-Clinical Adolescents

**DOI:** 10.1007/s10826-018-1099-z

**Published:** 2018-05-04

**Authors:** Peter Muris, Myrr van den Broek, Henry Otgaar, Iris Oudenhoven, Janine Lennartz

**Affiliations:** 1 Maastricht University, Maastricht, The Netherlands; 2 Stellenbosch University, Stellenbosch, South Africa; 3 City University of London, London, UK

**Keywords:** Self-compassion, Self-Compassion Scale, Positive and negative subscales, Psychopathology, Validity

## Abstract

To demonstrate that the positive and negative subscales of Self-Compassion Scale (SCS) are very different in nature, we conducted a series of face validity checks on the items of this questionnaire among psychologists and psychology students (Study 1). Furthermore, a survey was administered to a convenience sample of non-clinical adolescents to examine the relations between various SCS subscales and symptoms of anxiety and depression as well as coping styles (Study 2). The results of the face validity checks revealed that the positive subscales seem to be well in line with the protective nature of self-compassion as they were mainly associated with cognitive coping and healthy functioning, whereas the negative subscales were chiefly associated with psychopathological symptoms and mental illness. The survey data demonstrated that the positive SCS subscales were positively correlated with adaptive coping (*r*’s between .22 and .50) and negatively correlated with symptoms of anxiety and depression (*r*’s between −.19 and −.53), while the negative subscales were positively correlated with symptoms (*r*’s between .49 and .61) and maladaptive coping strategies such as passive reacting (*r*’s between .53 and .56). Additional analyses indicated the negative subscales of the SCS accounted for a significant proportion of the variance in symptoms, whereas the unique contribution of the positive SCS subscales was fairly marginal. We caution to employ the total SCS score that includes the reversed negative subscales as such a procedure clearly inflates the relation between self-compassion and psychopathology.

## Introduction

Over the past 25 years, psychological science has witnessed an explosive rise of positive psychology. This research domain is chiefly concerned with the study of positive emotions and positive character traits, and their role in the promotion of physical and psychological well-being (Fredrickson and Losada 2005; Seligman et al. 2005). Self-compassion is a concept that seems to fit well under the umbrella of positive psychology; it refers to the tendency of maintaining a positive attitude towards oneself, when facing personal shortcomings, inadequacies, and failures. Neff (2003a), who is one of the leading scholars in this field, has initially defined the construct as containing three components: (1) self-kindness, which refers to the tendency to be caring and understanding with oneself when confronted with personal adversity rather than engaging in harsh self-criticism and self-judgment; (2) common humanity, which concerns the inclination to recognize that personal failures and problems are a normal part of human life rather than viewing such experiences as evidence for being separated and isolated from other people; and (3) mindfulness, which is defined as the ability to keep one’s difficulties and associated negative feelings in balanced awareness rather than becoming too absorbed and over-identified with them.

To measure individual differences in self-compassion, Neff (2003b) constructed the Self-Compassion Scale (SCS), a 26-item questionnaire that measures the three core components of self-kindness, common humanity, and mindfulness as well as their “negative” counterparts of self-judgment, isolation, and over-identification. The SCS yields a total score that combines the three core components and the reversely scored counterparts, which is reliable in terms of internal consistency and test-retest stability. The SCS has been widely applied in clinical and health psychology research to investigate the protective influence of self-compassion on psychological well-being. In general, this research has noted that self-compassion is negatively associated with anxiety, depression, stress, and other mental health issues, underscoring the positive nature of the trait (MacBeth and Gumley 2012).

In the meantime, critique has been raised regarding the validity of the SCS (Muris et al. 2016). This criticism has mainly centered on the “negative” components of self-compassion (i.e., self-judgment, isolation, and over-identification), and their inclusion in the total score of the scale. For example, in a meta-analysis conducted by Muris and Petrocchi (2017) investigating the relations between the SCS subscales and psychopathology, it was found that the positive components of self-compassion were negatively associated with mental health problems, which confirmed their hypothesized protective nature. In contrast, the negative components were positively linked to psychopathology, suggesting that these subscales tap increased vulnerability to mental health problems. Furthermore, tests for comparing the strength of the relations between various SCS subscales and psychopathological symptoms indicated that the negative components were significantly stronger associated with mental health problems than the positive components. This finding led the authors to conclude that the use of the SCS total score, which typically includes the reversely scored negative subscales, will probably result in an inflated negative relationship between self-compassion and symptoms of psychopathology. As such, it was argued that the total SCS score should not include the negative subscales.

This notion was further empirically tested by Muris (2016) who administered the shortened version of the SCS (Raes et al. 2011) that also assesses negative and positive components of self-compassion, and Achenbach’s (2009) Youth Self-Report for measuring emotional and behavioral symptoms, in a sample of 184 non-clinical adolescents aged 12–16 years. As predicted, it was found that the positive components of self-compassion were negatively and the negative components were positively related to symptom levels. Yet, again the relations between negative components and symptoms were considerably stronger than those observed between positive components and symptoms. Most importantly, additional analyses showed that the percentage of explained variance in symptoms for the negative components of self-compassion was three to five times larger than that for the positive components. Muris (2016) concluded that the negative subscales of the SCS appear to tap a number of toxic mechanisms that do not fit with the true protective nature of self-compassion, and that their inclusion in the total score will magnify the (negative) link with psychopathology.

A comparable conclusion was drawn by Pfattheicher et al. (2017) who examined the relation between the negative and positive components of self-compassion as measured with the SCS and neuroticism, which is the personality trait that reflects a person’s susceptibility for experiencing negative emotional states and is generally associated with heightened levels of psychopathology (Ormel et al. 2013). The researchers documented extremely high positive correlations between the negative subscales of the SCS and neuroticism (see also Lopez et al. 2015), and hence concluded that these self-compassion components are completely redundant with this vulnerability personality trait.

In response to these critiques, Neff (2016a, 2016b) argued that the SCS is the most optimal measure for assessing self-compassion in the way she has theoretically defined the construct, namely as a balance between the compassionate features of self-kindness, common humanity, and mindfulness, and the negative characteristics of self-judgment, isolation, and over-identification. To substantiate her argument, she referred to recently conducted factor analytic studies (Claire et al. 2018; Neff et al. 2017), which showed that the SCS is best represented by a bi-factor model, which assesses covariance between factors that arise from the presence of an overarching factor, in this case self-compassion, whilst allowing the individual factors (subscales) to retain and account for variance in their own subset of items. On the basis of these findings, Neff et al. (2017) argued that her scale measures self-compassion as a multifaceted construct, but that there is also sufficient justification for using the total score as a general index of self-compassion. While providing evidence for the construct validity of the SCS and supporting Neff’s (2003a) idiosyncratic definition of self-compassion, factor analytic research remains silent about other aspects of validity of the scale and especially its positive and negative components. In other words, the structure of an instrument can be fully in keeping with a theoretical notion (which means that the scale has good internal validity), but this does not necessarily imply that a scale actually measures what it intends to measure and hence has predictive value (i.e., external validity).

Self-compassion is a way of dealing with oneself when in pain or in trouble, and as such “can be conceptualized as a coping strategy that promotes well-being and positive psychological functioning” (Batts et al. 2010, p.108). The SCS components of self-kindness, common humanity, and mindfulness all seem to fit well with this conceptualization, but the negative components are less compatible with this notion. In fact, pure from a theoretical and definitional point-of-view, the negative components parallel psychopathological symptomatology. That is, self-judgment shows clear similarities with harsh self-criticism (Zuroff et al. 1990), isolation shares features with social withdrawal and loneliness (Rubin et al. 2004), and over-identification matches with self-absorption and self-focused rumination (Lyumbomirsky and Nolen-Hoeksema 2015). These are all clearly negative features and therefore it is not surprising that critics question their validity and have associated them with neuroticism and psychopathology (Muris 2016; Muris et al. 2016; Muris and Petrocchi 2017; Pfattheicher et al. 2017). However, Neff (2016a) has countered this critique by stating that the negative components of the SCS “are negative ways of relating to oneself that can *lead* to psychopathological outcomes … but are not *the same* as psychopathological outcomes … [and that] thus claims of tautology are not relevant” (p. 795). Critically, this remark implies that there is a direct causal chain between the negative components and psychopathology, while at the moment such cannot be concluded as available data are mostly correlational.

We continue the debate about this point and maintain that the negative components of the SCS have more in common with psychopathology than with coping. To further demonstrate the differential nature of the positive and negative components of self-compassion, we conducted a series of face validity checks of the SCS (Study 1) by asking two separate panels of psychologists and psychology students to categorize the items of this questionnaire (a) either as “cognitive coping” or as “psychological symptom,” and (b) as characteristic for a normal healthy or a clinically referred person. It was expected that SCS items belonging to the positive components of self-compassion would be more frequently categorized as “cognitive coping” and typical for a normal healthy person, whereas SCS items belonging to the negative components were hypothesized to be more often categorized as “psychological symptom” and characteristic for a clinically referred person. In addition, we administered a survey in a convenience sample of non-clinical adolescents (Study 2) to examine the relations between the positive and negative components of self-compassion as measured with the SCS on the one hand and symptoms of psychopathology (i.e., anxiety and depression) as well as coping styles on the other hand. Here, we predicted that the negative components of the SCS would be more intimately correlated with symptoms of anxiety and depression, while the positive components of the scale would be more closely connected to (positive) coping strategies. Further, we tested empirically whether the nature of the positive and negative components of self-compassion are indeed different by conducting a joint principal components analysis on the SCS subscales, symptoms, and coping strategies measures. We anticipated a two-component solution with the positive SCS subscales and adaptive coping strategies clustering on the one and the negative SCS subscales and symptoms clustering on the other factor. Finally, we used these data to demonstrate once again (see also Muris 2016) that the link between self-compassion and symptoms of anxiety and depression to a large extent is carried by the negative components of the SCS, and that inclusion of these components in the total score inflates the relation between self-compassion and these types of psychopathology.

Study 1: Face validity checks

## Method

### Participants

The face validity checks of the SCS items were carried out by two panels of psychology students and psychologists of Maastricht University. The first panel consisted of 21 psychology students (all females in their early 20s) of the research master in psychopathology, while the second panel was composed of 21 psychologists (16 females and 6 males aged between 20 and 30 years) who worked as Ph.D. student at the Department of Clinical Psychological Science and conducted their research in the domains of clinical psychology, behavioral medicine, or forensic psychology (none of them was involved in research on self-compassion).

### Procedure

Both panels received a list of the 26 SCS items (in the order that they appear in the original scale) along with a written instruction. The instruction given to the first panel was: “This is a small survey testing the validity of some items. Below you will find a list of 26 items: Some items are indicative of a psychological symptom, while other items are concerned with cognitive coping. Please indicate for each item whether you think that it pertains to a symptom scale or a coping scale.” The instruction provided to the second panel ran as follows: “This is a small survey testing the clinical relevance of some items. Below you will find a list of 26 statements: Some statements are from normal, healthy individuals who do not suffer from a psychological disorder, while other statements have been made by clinically referred individuals who show clear signs of at least one psychological disorder. Please indicate for each statement whether you think that it has been made by a normal, healthy person without a psychological disorder or whether it was made by a clinically referred individual with a psychological disorder. In case you indicate that the statement has been made by a person with a psychological disorder, please specify which disorder(s) you think of.” Participants in both panels were explicitly asked to indicate whether they were familiar with the items listed in the survey, but none of them indicated this to be the case.

## Results

The results of the first face validity check in which students were invited to classify SCS items as a psychological symptom or as a form of cognitive coping are displayed in Table 1. As can be seen, items belonging to the three positive components of self-kindness, common humanity, and mindfulness were predominantly viewed as a manifestation of cognitive coping (all percentages ≥ 80.94), whereas items belonging to the three negative components of self-judgment, isolation, and over-identification were predominantly regarded as psychological symptoms (all percentages ≥ 85.72).

**Table1:** Results of the first face validity check of the SCS: Right columns display average percentages of psychology students (*N* = 21) categorizing items of each subscale as psychological symptom or as cognitive coping

SCS subscale	Item example	Number of items	Subscale type	Psychological symptom	Cognitive coping
Self-kindness	I am kind to myself when I am experiencing suffering	5	Positive	14.28	85.72
Self-judgment	When times are really difficult, I tend to be tough on myself	5	Negative	80.94	19.06
Common humanity	I try to see my failings as part of the human condition	4	Positive	5.95	94.05
Isolation	When I fail at something important, I tend to feel alone in my failings	4	Negative	91.67	8.33
Mindfulness	When something upsets me I try to keep my emotions in balance	4	Positive	4.80	95.20
Over-identification	When something upsets me I get carried away with my feelings	4	Negative	83.35	16.65

SCS Self-Compassion Scale

Table 2 shows the results of the second face validity check in which we took a more extreme approach by asking psychologists to categorize various SCS items as characteristic for a normal healthy or a clinically referred person. Again, our expectations were confirmed by the data: that is, the items of the three positive components were mostly classified as statements made by a normal, healthy person (all percentages ≥ 85.72), while items of the three negative components were primarily categorized as statements of a clinically referred individual (all percentages ≥ 58.10). Note also that the psychologists most commonly linked items of self-judgment, isolation, and over-identification to mood, anxiety, eating, personality, and attention-deficit disorders.

**Table 2:** Results of the second face validity check of the SCS: Right columns display average percentages of psychologists (*N* = 21) categorizing items as characteristic for a normal healthy or a clinically referred person

SCS subscale	Subscale type	Normal, healthy person	Clinically referred person	Most frequently linked disorder(s)*
Self-kindness	Positive	85.72	14.28	Mood: 5.72
Self-judgment	Negative	41.90	58.10	Mood: 28.58, Pers: 23.80, Eat: 21.88, Anx: 15.26
Common humanity	Positive	94.02	5.98	−
Isolation	Negative	33.3	66.7	Mood: 45.23, Anx: 33.35, Pers: 11.90, Eat: 7.15, ADD: 7.15
Mindfulness	Positive	95.22	4.78	−
Over-identification	Negative	22.63	77.37	Anx: 61.93, Mood: 54.75, Pers: 21.43, Eat: 9.53, ADD: 7.15

SCS Self-Compassion Scale, Mood Mood Disorder, Pers Personality Disorder, Anx Anxiety Disorder, Eat Eating Disorder, ADD Attention-Deficit Disorder. * Only disorders are shown with an average percentage of >5%

## Discussion

These face validity checks are well in keeping with our principal argument that the SCS consists of two sets of items which are clearly distinct in terms of content. That is, half of the items seem to be in line with the protective nature of the self-compassion construct as they were mainly associated with cognitive coping and normal, healthy functioning. The other half of items, however, appear to be difficult to reconcile with a positive psychology concept as they were chiefly linked to psychological symptoms and mental illness. Interestingly, the negative subscales of the SCS (i.e., self-judgment, isolation, and over-identification) were frequently related to mood and anxiety disorders, which happen to be the types of psychopathology that have most often been the topic of investigation in self-compassion research (MacBeth and Gumley 2012; Muris and Petrocchi 2017). In our conviction, the inclusion of the negative components in the SCS will inflate the relation between self-compassion and symptoms of anxiety and depression and thus will produce misleading and invalid information on the relevance of this protective feature within the context of these mental health problems. We will empirically examine this point in Study 2.

Study 2: Negative and positive components of self-compassion, coping, and symptoms

## Method

### Participants

One-hundred-and-thirty high school students (44 boys and 86 girls) of Raayland College in Venray, the Netherlands, participated in this study. The mean age of the youngsters was 16.68 years (SD = .89, range: 15–19 years). The participants were recruited in two educational levels, higher general secondary education (49.2%) and pre-university education (50.8%).

### Procedure

An information letter about the purpose and procedure of the study was sent to the director of the school to ask permission for conducting the survey in the grades 4 to 6. Following permission, the students (*N* = 800) and their parents received information letters and consent forms. Those students who handed in a signed informed consent form (16.25%) completed the set of questionnaires at school during Dutch language classes. Cinema tickets were raffled among the students who participated in this study. The research project was approved by the Ethical Review Committee Psychology and Neuroscience (ERCPN) at Maastricht University.

### Assessment

As already described in the introduction, the SCS is a self-report questionnaire for measuring self-compassion (Neff 2003a). The scale consists of 26 items, half of which can be allocated to the three positive subscales of self-kindness (5 items; e.g., “I try to be loving towards myself when I am feeling emotional pain”), common humanity (4 items; e.g., “When things are going badly for me, I see the difficulties as part of life that everyone goes through”), and mindfulness (4 items; e.g., “When something upsets me I try to keep my emotions in balance”), and half of which belong to the three negative subscales of self-judgment (5 items; e.g., “I am disapproving and judgmental about my own flaws and inadequacies”), isolation (4 items; e.g., “When I think about my inadequacies, it tends to make me feel more separate and cut off from the rest of the world”), and over-identification (4 items; e.g., “When I am feeling down I tend to obsess and fixate on everything that is wrong”). Responses are given on a five-point scale ranging from 1 (almost never) to 5 (almost always). Subscale scores can be computed by summing the ratings on relevant items and a total self-compassion score can be obtained by combining the ratings on all items, after reversely coding responses on the items of the negative subscales (i.e., self-judgment, isolation, and over-identification). Reliability coefficients that were found in the current study were well in line with those that have been reported previously, with Cronbach’s alphas being .89 for the total score and ranging between .61 and .84 for the subscales.

The trait anxiety version of the *State-Trait Anxiety Inventory for Children* (STAI-C; Spielberger 1973) is a 20-item scale for measuring chronic symptoms of anxiety, worry, and stress. Children and adolescents are asked to rate the frequency with which they experience anxiety symptoms such as “I am scared.” “I feel troubled,” and “I get a funny feeling in my stomach” using three-point scales: 1 = almost never, 2 = sometimes, and 3 = often. A total trait anxiety score can be calculated by summing the ratings on all items. The STAI is a widely used scale for measuring anxiety symptoms in youngsters and its psychometric properties are well-established (Silverman and Ollendick 2005). In the present investigation, the STAIC displayed good reliability, with an alpha coefficient of .88.

The *Children’s Depression Inventory* (CDI; Kovacs 1981) is a commonly employed self-report measure of depressive symptoms in children and adolescents. The scale has 27 items dealing with sadness, self-blame, loss of appetite, insomnia, interpersonal relationships, and school adjustment. Sample items are “I am sad all the time,” “Most of the days, I am not hungry,” and “I feel like crying every day.” CDI items are scored on three-point scales (0 = not true, 1 = somewhat true, 2 = very true). A total CDI score can be calculated by summing all item scores and varies between 0 (no depression symptoms) and 54 (all depression symptoms clearly present). The psychometric properties of the CDI have been tested extensively and are found to be adequate in clinical and non-clinical samples of children and adolescents (e.g., Saylor et al. 1984). In this study, internal consistency proved to be excellent, with a Cronbach’s alpha of .92.

The *Utrecht Coping List for Adolescents* (UCL-A; Bijstra et al. 1994) is a self-report questionnaire for measuring various coping styles of adolescents. Originally, the questionnaire was developed for adults (Schreurs et al. 1993), but the questionnaire was revised to make it applicable to the adolescent population. The UCL-A consists of 47 items, in the form of descriptive statements, which can be allocated to seven subscales: active tackling (7 items; e.g., “I immediately do something about the problem”), palliative reacting (8 items; e.g., “I try to be calm”), avoidance (8 items; e.g., “I try to avoid the problem”), social support seeking (6 items; e.g., “I share my worries with someone”), passive reacting (7 items; e.g., “I can think of nothing more than the problem”), expression of emotions (3 items; e.g., “I show that I am angry with the person who caused the problem”), and reassuring thoughts (5 items; e.g., “I tell to myself that everything will turn out well”). Active tackling, palliative reacting, social support seeking, and reassuring thoughts are considered as adaptive coping strategies, while avoidance, passive reacting, and expression of emotions can be regarded as less functional (Schreurs et al. 1993). UCL-A items are rated on a four-point Likert scale (1 = rarely or never, 2 = sometimes, 3 = often, and 4 = very often) reflecting the extent to which adolescents judge the statements as applicable to them. The psychometric qualities of the scale have not been extensively investigated but available evidence supports the reliability and validity of the scale (Mavroveli et al. 2007; Meijer et al. 2002; Schreurs et al. 1993; Turner et al. 2012). In the current study, Cronbach’s alphas were .82 for active tackling, .64 for palliative reacting, .60 for avoidance, .90 for social support seeking, .78 for passive reacting, .70 for expressing emotions, and .56 for reassuring thoughts.

## Results

### Correlations between positive and negative SCS subscales and symptoms and coping strategies

Table 3 displays correlations between various SCS subscales and measures of anxiety (STAIC), depression (CDI), and coping strategies (UCL-A). The correlations between the SCS subscales and the symptom measures showed a consistent pattern: the positive subscales of self-kindness, common humanity, and mindfulness were all negatively correlated with symptoms of anxiety and depression (*r*’s between −.19 and −.53, *p* < .05), which indicates that higher levels of these self-compassion components were associated with lower levels of these emotional symptoms. The reverse was true for the negative subscales: that is, self-judgment, isolation, and over-identification were all positively correlated with anxiety and depression (*r*’s between .49 and .61, all *p* < .001), signifying that higher levels on these self-compassion counterparts were associated with higher symptom levels.

**Table 3:** Correlations among the SCS subscales and the other questionnaires

	SCS					
	Self-kindness	Self-judgment	Common humanity	Isolation	Mindfulness	Over-identification
STAIC Anxiety	−.38_a_**	.61_b_**	−.19_a_*	.58_b_**	−.25_a_*	.58_b_**
CDI Depression	−.53_a_**	.58_a_**	−.32_a_**	53_b_**	−.38_a_**	.49_a_**
UCL-A						
Active tackling	.50_a_**	−.25_b_*	.37_a_**	−.25_a_*	.48_a_**	−.25_b_*
Palliative reacting	.07_a_	.18_a_*	.13_a_	−.02_a_	.22_a_*	.06_a_
Avoidance	−.07_a_	.19_a_*	.00_a_	.22_b_*	−.09_a_	.09_a_
Social support seeking	.42_a_**	−.25_b_*	.22_a_*	−.28_a_*	.30_a_*	−.08_b_
Passive reacting	−.47_a_**	.56_a_**	−.33_a_**	.53_b_**	−.36_a_**	.56_b_**
Expression of emotion	−.29_a_*	.25_a_*	−.23_a_*	.17_a_	−.21_a_*	.26_a_*
Reassuring thoughts	.41_a_**	−.11_b_	.47_a_**	−.09_b_	.35_a_**	−.19_a_*

*N* = 130. SCS Self-Compassion Scale, STAIC State-Trait Anxiety Inventory for Children, CDI Children’s Depression Inventory, UCL-A Utrecht Coping List for Adolescents. Correlations not sharing similar subscripts signify that the strength of the associations with symptom and coping measures was significantly different for self-kindness vs. self-judgment, common humanity vs. isolation, and mindfulness vs. over-identification. *p < .05, **p < .001

The correlations between the SCS subscales and coping strategies were largely as anticipated. The positive components of self-kindness, common humanity, and mindfulness were positively correlated with adaptive coping strategies such as active tackling, social support seeking, and reassuring thoughts (*r*’s between .22 and .50, *p* < .05) and negatively correlated with maladaptive coping styles such as passive reacting and expression of emotion (*r*’s between −.21 and −.47, *p* < .05). The negative components of self-judgment, isolation, and over-identification were substantially and positively correlated with passive reacting (*r*’s between .53 and .56, *p* < .001). Other correlations between these negative SCS subscales and coping styles were smaller and showed a less consistent pattern, but the significant correlations generally indicated positive links with maladaptive strategies and negative links with adaptive styles.

To further demonstrate the differential pattern in the correlations involving the positive and negative SCS subscales, we also conducted tests for comparing correlated correlation coefficients (Meng et al. 1992). More precisely, we compared the strength of the correlations with psychopathological symptoms and coping styles for self-kindness vs. self-judgment, common humanity vs. isolation, and mindfulness vs. over-identification (to make these comparisons possible, the negative components of the SCS were reversed—as is also done when computing a total score for the SCS; see Muris and Petrocchi 2017). As shown in Table 3, the statistically significant differences that emerged were all in line with our hypothesis. That is, the negative subscales of self-judgment, isolation, and over-identification were significantly stronger associated with symptoms of anxiety and depression than their positive counterparts self-kindness, common humanity, and mindfulness (4 out of 6 comparisons). The reverse was true for the correlations involving adaptive coping styles: the positive SCS subscales were significantly stronger related to strategies such as active tackling, social support seeking, and reassuring thoughts than the negative SCS subscales (6 out of 9 comparisons).

### Factor analysis of SCS subscales, symptoms and coping measures

To empirically test the differential nature of the positive and negative components of self-compassion, we conducted a joint factor analysis on the SCS subscales, STAI, CDI, and UCL subscales. The adequacy of performing such test was confirmed by the value of the Kaiser-Meyer-Omin measure (.83) and Bartlett’s test of sphericity [*χ*^2^(105) = 926.07, *p* < .001]. Based on the criteria described by Field (2009), the extraction of the anticipated two factors was feasible (the two factors had Eigenvalues of 5.57 and 2.23 and explained together 52.03% of the total variance). Table 4 shows the loadings of various (sub)scales on the two factors. As can be seen, the result of this analysis was well interpretable and largely as anticipated. That is, the first factor consisted of the emotional symptoms scales (anxiety and depression), a number of maladaptive coping strategies (passive reacting, avoidance, and expression of emotion), and the negative SCS subscales of self-judgment, isolation, and over-identification. The second factor was composed of the three positive SCS subscales of self-kindness, common humanity, and mindfulness and a number of what can be considered as adaptive coping strategies (reassuring thoughts, active tackling, palliative reacting, and social support seeking). There were few substantial secondary loadings; a clear exception was palliative reacting which loaded positively on both factors. Palliative reacting is concerned with mental disengagement (avoiding thoughts about the stressor; see Turner et al. 2012), which, although originally intended as a form of adaptive coping (Schreurs et al. 1993), can be regarded as having positive as well as negative features.

**Table 4:** Results of the joint factor analysis (principal components with Varimax rotation, forced to extract two factors) performed on the SCS subscales and psychopathology and coping scales

	Factor 1	Factor 2
STAIC Anxiety	**.85**	−.13
UCL-A Passive reacting	**.83**	−.23
CDI Depression	**.81**	−**.31**
SCS Self-judgment	**.79**	−.18
SCS Isolation	**.73**	−.13
SCS Over-identification	**.73**	−.16
UCL-A Avoidance	**.42**	.07
UCL-A Expression of emotion	**.31**	−.23
SCS Mindfulness	−.21	**.75**
SCS Common humanity	−.06	**.73**
SCS Self-kindness	−**.41**	**.72**
UCL-A Reassuring thoughts	.05	**.72**
UCL-A Active tackling	−**.35**	**.57**
UCL-A Palliative reacting	**.39**	**.49**
UCL-A Social support seeking	−.24	**.48**

*N* = 130. SCS Self-Compassion Scale, STAIC State-Trait Anxiety Inventory for Children, CDI Children’s Depression Inventory, UCL-A Utrecht Coping List for Adolescents. Loadings >.30 are printed in bold

### Negative SCS subscales increase the link between self-compassion and symptoms

**Fig. 1:**
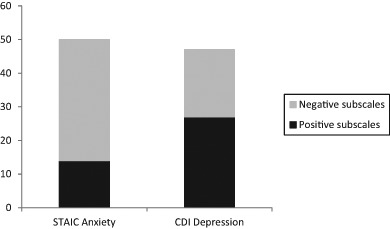
Percentages of explained variance in symptoms of anxiety and depression accounted for by the negative and positive subscales of the SCS. SCS Self-Compassion Scale

Hierarchical regression analyses were carried out to test the notion that the inclusion of the negative SCS subscales will increase the relation between self-compassion and symptoms of anxiety and depression. In these analyses, STAIC and CDI scores were the dependent variables, while the positive subscales of self-kindness, common humanity, and mindfulness (entered into the equation on Step 1) and the negative subscales of self-judgment, isolation, and over-identification (added to the model on Step 2) were the predictors. As can be seen in Table 5, on Step 1, the positive SCS subscales together explained a significant percentage of the variance in symptoms of anxiety (i.e., 14%) and depression (27%) on Step 1. In particular, self-kindness was found to be a relevant, negative predictor of both types of symptoms: higher levels of this self-compassion component were associated with lower levels of anxiety and depression. When entering the negative SCS subscales on Step 2, the percentages of explained variance significantly increased for both types of symptoms (anxiety: with 36% to a total of 50%; depression: with 20% to a total of 47%; see also Fig. 1). In the case of anxiety symptoms, all negative subscales made unique and significant positive contributions: thus, higher levels of self-judgment, isolation, and over-identification were accompanied by higher levels of anxiety. In the case of depression, only the negative subscale of isolation made an independent and significant positive contribution: again higher levels of this negative self-compassion component were associated with higher levels of depression.

**Table 5:** Results of the hierarchical regression analyses in which symptoms of anxiety (top panel) and depression (bottom panel) were explained from positive (step 1) and negative (step 2) SCS subscales

	*B*	SE	β	*R* ^2^
STAIC Anxiety				
Step 1				.14**
SCS Self-kindness	−4.29	1.27	−.38**	
SCS Common humanity	.99	1.06	.09	
SCS Mindfulness	−.56	1.35	−.05	
Step 2				.50**
SCS Self-kindness	−.08	1.18	−.01	
SCS Common humanity	.27	.84	.03	
SCS Mindfulness	−.67	1.05	−.06	
SCS Self-judgment	2.60	.98	.28*	
SCS Isolation	2.28	.76	.26*	
SCS Over-identification	2.41	.86	.25*	
CDI Depression				
Step 1				.27**
SCS Self-kindness	−5.68	1.23	−.48**	
SCS Common humanity	.05	1.03	.00	
SCS Mindfulness	−.82	1.31	−.07	
Step 2				.47**
SCS Self-kindness	−2.44	1.28	−.21	
SCS Common humanity	−.50	.90	−.04	
SCS Mindfulness	−.91	1.13	−.07	
SCS Self-judgment	2.09	1.06	.22	
SCS Isolation	2.38	.82	.26*	
SCS Over-identification	1.14	.93	.11	

*N* = 130. SCS Self-Compassion Scale, STAIC State-Trait Anxiety Inventory for Children, CDI Children’s Depression Inventory. *p < .05, **p < .001. For both regression analyses, no problems of multicollinearity were detected; all tolerance values were >.36 and VIF values <3

## Discussion

The results of Study 2 confirmed our hypothesis that there is a differential pattern of correlations between the negative and positive components of self-compassion on the one hand, and psychopathological symptoms and coping styles, on the other hand. More precisely, it was found that the negative SCS subscales were positively correlated with symptoms of anxiety and depression, and these correlations were in most cases also significantly stronger than the inverted correlations observed between the positive SCS subscales and these symptoms. This result mirrors Muris and Petrocchi’s (2017) meta-analytic findings that self-judgment, isolation, and over-identification were all substantially and positively correlated with psychopathological symptoms, which made these authors conclude that these SCS subscales are likely to tap an increased vulnerability to mental health problems (Lopez et al. 2015; Montero-Marin et al. 2016). This of course also fits nicely with Pfattheicher et al. (2017) conclusion that the negative SCS components are completely redundant with neuroticism, the personality factor characterized by a heightened susceptibility to experience negative emotions and an increased proneness to develop mental health problems.

The relations between self-compassion and coping were well in line with the theoretical notions of Batts Allen and Leary (2010). The positive SCS subscales were all positively associated with adaptive coping styles such active tackling, social support seeking, and reassuring thoughts. Further, the negative SCS subscales were negatively correlated with adaptive coping strategies (although these correlations were often significantly weaker than those between positive SCS subscales and adaptive strategies) and to some extent positively correlated with maladaptive strategies such as passive reacting, avoidance, and expression of emotion. This general pattern of findings is also in line with an empirical study by Sirois et al. (2015) who investigated the relationship between self-compassion and coping in two samples of participants suffering from a chronic illness (i.e., inflammatory bowel disease and arthritis). Although the researchers did not differentiate between the negative and positive components of the SCS, total self-compassion appeared to be positively linked to adaptive coping strategies (e.g., active coping, planning, and positive reframing) and negatively related to maladaptive strategies (e.g., behavioral disengagement and self-blame).

While the correlations with symptom and coping measures already indicated that the positive and negative subscales of the SCS exhibit divergent validity, we also conducted a joint factor analysis on all questionnaire data to further harden this point. As anticipated, a two-factor solution was found with the positive SCS subscales loading on a factor that was also composed of adaptive coping styles (i.e., reassuring thoughts, active tackling, and support seeking) and the negative SCS subscales significantly loading on a factor that further consisted of psychopathological symptoms (i.e., anxiety and depression) and maladaptive coping strategies (i.e., passive reacting, avoidance, and expression of emotion). These results once more demonstrate that the positive and negative components of self-compassion are quite different in nature.

The results of Study 2 further replicated the findings of Muris (2016) by demonstrating that the negative subscales of the SCS have an important share in the relation between self-compassion and psychopathological symptoms. More precisely, it was found that the negative subscales of the SCS explained almost three times more of the variance in anxiety symptoms than the positive subscales. In the case of depression, the contributions of the negative and positive subscales were more in balance, with each accounting for about half of the variance in this type of symptoms. In any case, when taking into account that the negative components have so much in common with psychopathology in the first place, it is difficult to evade the conclusion that studying the link between the negative SCS subscales and psychopathological symptoms is a tautological exercise and that the inclusion of these subscales in a SCS total score will obviously inflate the relationship between self-compassion and psychopathology.

## General discussion

Although it should be acknowledged that both studies suffer from limitations (Study 1: we could have employed an open question rather than a forced choice format to assess the face validity of the SCS; Study 2: the reliability of some UCL-C coping scales was insufficient), the results indicate that the SCS, the commonly employed and popular scale for measuring self-compassion, is not the most optimal instrument for assessing this protective construct (Muris 2016; Muris et al. 2016). That is, face validity checks (Study 1) and empirical tests (Study 2) clearly revealed that the negative and positive subscales have a quite different character. The positive subscales are indicative for a healthy attitude towards oneself and can best be qualified as adaptive coping. They represent the good sides of self-compassion and reflect the true nature of this protective factor, which of course fits nicely within a positive psychology framework. In contrast, the negative subscales are concerned with an unwholesome attitude towards oneself and are fused with symptoms of psychopathology. They can be regarded as the bad sides of self-compassion, and are better removed from the SCS as they are indicators of vulnerability and emotional problems rather than protection (Muris and Petrocchi 2017).

In a previous paper, Neff (2016b) noted that “the assertion that use of a total SCS score inflates the link between self-compassion and psychopathology is a serious one [but] is in fact an empirical question.” We certainly agree with this notion, and in Study 2 we tested the merits of the total SCS score. The results (as well as those reported by Muris 2016) clearly indicated that the inclusion of the negative subscales in the total score indeed magnify the relation with symptoms of anxiety and depression. Unfortunately, most researchers show too little awareness of this problem; they continue to use the SCS total score that is composed of both the positive and the negative subscales and seem to be ignorant of the fact that this scoring method inflates the effect sizes of their findings. Yet, if one is really interested in the protective nature of self-compassion, one should no longer rely on a total score that includes the negative subscales. Another strategy could be to at least report on the separate relations between the positive and negative components and psychopathology, so that researchers can actually inspect the presence of an inflation effect.

So far, Neff (2016a, 2016b) has maintained that the inclusion of the negative components of self-judgment, isolation, and over-identification in the SCS is justified because this is in accordance with her original definition of self-compassion. We have good reasons to designate Neff’s (2003a, 2003b) original definition of self-compassion as an unfortunate one. Besides the present findings, there is an increasing number of recent empirical studies showing that the positive and negative subscales of the SCS are totally different by nature (e.g., Brenner et al. 2017; Coroiu et al. 2018; Lopez et al. 2018). Most importantly, the uniqueness of self-compassion lies in its protective nature represented in the three positive components, which makes this concept of special relevance within a context of mental health problems. The negative components are redundant in that they represent a number of maladaptive mechanisms that are obviously associated with neuroticism and psychopathology, and as such have already been (in our view: rightly) described as “old wine in new bottles” (Pfattheicher et al. 2017, p. 160). We think that the time is ripe to openly acknowledge this to the field, so that researchers can really start to focus on the protective nature of self-compassion.
